# Mechanical and Self-Healing Performance of Cement Composites Containing Bacteria Extracted from Waste Concrete

**DOI:** 10.3390/ma18245483

**Published:** 2025-12-05

**Authors:** Se-Jin Choi, Jeong-Yeon Park, Jung-Mi Kim, Ha-Yeon Song, Jae-In Lee

**Affiliations:** 1 Department of Architectural Engineering, Wonkwang University, Iksan 54538, Republic of Korea; csj2378@wku.ac.kr (S.-J.C.); jkite7490@naver.com (J.-Y.P.); 2 Department of Biomedical Materials Science, Jeonbuk Advanced Bio-Convergence Academy, Wonkwang University, Iksan 54538, Republic of Korea; micro@wku.ac.kr; 3 Department of Life and Environmental Science, Institute of Life Science and Natural Resources, Wonkwang University, Iksan 54538, Republic of Korea

**Keywords:** self-healing, *Lysinibacillus fusiformis*, waste concrete, mortar, compressive strength

## Abstract

**Highlights:**

**What are the main findings?**

- *Lysinibacillus fusiformis* solution (LFS) increased mortar flow, compressive strength development.
- LFS decreased the carbonation depth through densification and inhibited CO_2_ influx.
- LFS induced self-healing, showing an excellent compressive strength recovery rate.

**What are the implications of the main findings?**

- *L. fusiformis* overcomes the limitations of previous bacteria tested in highly alkaline concrete.
- Self-healing can mitigate the long-term damage to concrete structures.
- The durability and self-healing capacity will increase the service life of concrete structures.

**Abstract:**

Cracks can reduce the durability of concrete structures. To mitigate the damage caused, self-healing technologies using bacteria and cement-based materials can be utilized. For self-healing, bacteria contained within the matrix are advantageous because they can heal cracks upon introducing oxygen and water under favorable conditions. To our knowledge, this is the first study showing that *Lysinibacillus fusiformis* isolated from waste concrete induces calcite precipitation in a cement-based material. Replacing 5–20% of the mixing water with the bacterial solution increased mortar flow, and the initial compressive strength increased with the bacterial content. After long-term aging, the compressive strength of the sample with 20% bacterial solution was ~45.6 MPa, the highest among all samples. In terms of durability, the bacterial solution reduced the carbonation depth compared with that of a control sample without added bacteria, and the 20% sample showed 53% higher carbonation resistance than the control. In terms of the self-healing performance, the bacteria-loaded samples showed higher compressive strength recovery rates than the control sample, with the 20% sample showing the highest rate of approximately 131%. Therefore, *L. fusiformis* derived from waste concrete is a promising candidate bacterium for enhancing the durability and self-healing efficiency of cement composites.

## 1. Introduction

Concrete is widely used in various industries owing to its excellent compressive strength and durability [1,2]. However, because of its low tensile strength and high brittleness, along with various external factors, cracks inevitably form. These cracks enable harmful substances, such as chloride ions and carbon dioxide, to enter the concrete structure, which has been shown to reduce its strength and durability [3,4,5,6]. Methods that include injecting epoxy into concrete cracks or filling them with repair materials after V-cutting are used to prevent deterioration. However, these methods address cracks only after they have formed and necessitate repeated repairing if the filler material deteriorates over time. Furthermore, while self-healing through unhydrated cement particles is possible, this method is only effective for very small cracks, and the limited presence of unhydrated cement particles often leads to minimal self-healing.

In recent years, various self-healing cement composites have been reported to heal cracks occurring on the surface or inside cement composites without any additional processes or reconstruction procedures. These studies simply applied self-healing materials with the ability to repair cracks to mitigate the deteriorating strength and durability of concrete [7,8,9,10,11]. In the case of self-healing cement composites, studies have utilized bacteria, polymers, and cementitious materials, depending on the material used [12,13,14,15]; these methods are known to improve the durability of concrete by preventing leakage and blocking harmful substances from entering, thereby increasing the lifespan of concrete and reducing maintenance costs [16,17,18,19].

Among the methods for applying self-healing materials, self-healing cement composites containing bacteria have been shown to improve the density and strength of the matrix by precipitating crystalline calcium carbonate outside the cells through the biomineralization action of bacteria attached to the crack surface. This process, which is known as microbially induced calcite precipitation (MICP), fills microcracks and voids in the cement composite, thereby densifying and strengthening the matrix [20,21,22,23].

Bacterially promoted biomineralization induces the formation of calcium carbonate (CaCO_3_) through various metabolic pathways. Microorganisms that facilitate carbonate mineralization are generally categorized into three major groups: (i) ureolytic bacteria that produce urease, (ii) aerobic bacteria capable of converting organic carbon into inorganic carbon, and (iii) anaerobic denitrifying bacteria. Urea is converted into ammonia and carbon dioxide by the urease produced by ureolytic bacteria, with the ammonia subsequently increasing the local pH through the formation of hydroxide ions that react with carbon dioxide to yield bicarbonate and carbonate ions [7,8]. Ultimately, calcium ions present in the environment combine with the carbonate ions and precipitate as CaCO_3_ crystals, as represented by the following sequence:

(1) $${\left({NH}_{2}\right)}_{2}CO\;\left(aq\right)+H_{2}O\;\left(l\right)\overset{urease}{\to }{2NH}_{3}\left(aq\right)+{CO}_{2}(aq)$$

(2) $${NH}_{3}\left(aq\right)+H_{2}O\left(l\right)\to {NH}_{4}^{+}\left(aq\right)+{OH}^{-}(aq)$$

(3) $${CO}_{2}\;\left(aq\right)+{OH}^{-}\left(aq\right)\to {HCO}_{3}^{-}(aq)$$

(4) $${HCO}_{3}^{-}\;\left(aq\right)+{OH}^{-}\left(aq\right)\to {CO}_{3}^{2-}\left(aq\right)+H_{2}O\left(l\right)$$

(5) $${Ca}^{2+}\;\left(aq\right)+{CO}_{3}^{2-}\left(aq\right)\to {CaCO}_{3}\;(s)↓$$

In addition, spore-forming bacteria such as those belonging to the *Bacillus* and *Clostridium* genera are able to survive under extreme environmental conditions by producing dormant endospores. These endospores remain structurally intact yet metabolically inactive for extended periods, even in the absence of essential elements, such as carbon sources, nutrients, moisture, and oxygen. Cracks expose the dormant spores to favorable conditions; consequently, they germinate and reinitiate metabolic activity, thereby inducing MICP and contributing to autonomous crack healing [12].

Bacteria can also hibernate as endospores in the healed area, which can be expected to have a secondary crack-healing effect when cracks reform in the future [24,25,26,27].

However, when bacteria are incorporated into cement composites in live form without any additional treatment, the survival rate of bacteria is reduced to approximately 90% or less in the high-pH environment of cement composites, which makes it difficult for bacteria to survive [28,29,30].

Research aiming to overcome this poor survival rate and to exploit the potential for calcium carbonate precipitation through the MICP action of bacteria is currently underway. One strategy is to incorporate bacteria that are viable in high pH environments into aqueous solutions. Alternatively, bacteria have been encapsulated in porous lightweight aggregates, such as expanded clay and expanded vermiculite, and in polymers such as alginates and hydrogels. These capsules are then incorporated into cement composites [31,32,33,34].

For example, Pourfallahi et al. [31] evaluated the self-healing performance of concrete incorporated with bacteria isolated from alkaline soil in the form of an aqueous solution. They reported that Portland pozzolanic cement and Portland cement type 2 samples incorporating *Bacillus* sp. showed relatively higher healing performance than the control samples. Vigay et al. [32] evaluated the healing performance of concrete with various types of bacteria and found that *B. pasteurii* and *B. subtilis* showed relatively better crack healing than the other bacteria. Skevi et al. [33] reviewed the effects of live and dead bacterial cells on the mechanical performance of cement mortars and demonstrated that both live and dead bacteria could increase the strength of cement composites. In the case of dead bacteria, they provided nucleation sites that promoted hydrate production.

Riad et al. [34] evaluated the healing performance of concrete based on the type and number of bacteria (*Sporosarcina pasteurii*, *B. sphaericus*) incorporated.

Their results showed that regardless of the type of bacteria, the samples with 10% bacteria showed relatively good healing strength, which was attributed to the filling of microcracks due to the precipitation of calcium carbonate, which was confirmed by scanning electron microscopy (SEM) and energy dispersive X-ray spectroscopy.

Although a number of studies have incorporated bacteria such as *Sporosarcina* and *Bacillus* spp. isolated from highly alkaline environments into concrete, either encapsulated or in aqueous solution, reports involving *Lysinibacillus* spp. are limited. While previous studies have shown that *Sporosarcina pasteurii* and *Bacillus subtilis* are effective MICP agents owing to their high urease activities and spore-forming abilities, they also present limitations, including ammonia overproduction and relatively low tolerances to highly alkaline cementitious environments [35,36]. Recent reports suggest that *L. fusiformis* may overcome some of these drawbacks because it adapts strongly to saline–alkaline conditions while maintaining effective urease activity [37]. Recent studies [37,38] have revealed the use of *L. fusiformis* isolated from soil for use in MICP applications, primarily focusing on soil stabilization and tailing sand remediation. Members of the *Lysinibacillus* genus are commonly isolated from waste environments and are known for their abilities to utilize minerals. Calcium carbonate is reportedly precipitated in rare cases, with *L. sphaericus* a notable example [39], which suggests that *Lysinibacillus* spp. are also potential candidates for use in concrete self-healing and repair applications.

However, to our knowledge, no studies have reported the isolation of *L. fusiformis* from waste concrete or evaluated its applicability to cement-based materials. Accordingly, we investigated the mechanical properties, durabilities, and healing behavior of cement composites that incorporate *L. fusiformis* isolated from waste concrete.

## 2. Materials and Methods

### 2.1. Materials

The cement used in this study was a type 1 ordinary Portland cement (Sampyo Cement, Seoul, Republic of Korea) with a density of 3.13 g/cm^3^ and a Blaine fineness of 3820 cm^2^/g, as shown in Table 1. Natural sand with a density of 2.60 g/cm^3^ and a fineness modulus of 2.45 was also used.

*L. fusiformis* A15 (KCTC16263BP) was the microbial strain used in this study; it was isolated from waste concrete and deposited at the Korean Collection for Type Cultures (KCTC, Jeongup, Republic of Korea). The morphology and physiological characteristics of *L. fusiformis* were verified by optical and electron microscopy (Leica ICC5-E, Wetzlar, Germany) prior to each MICP experiment.

In particular, scanning electron microscopy was performed using a field-emission SEM (Hitachi S-4800, Tokyo, Japan). Samples were fixed, dehydrated through a graded ethanol series, dried, and coated with platinum using a sputter coater prior to observation. Imaging was conducted at an accelerating voltage of 5–15 kV under high-vacuum conditions.

The following equations represent the reaction equations for calcium carbonate precipitation by bacteria incorporated inside the cement composite [40]:

Ca^2+^ + Bacterial cell → Cell-Ca^2+^ (6)

Cell-Ca^2+^CO_3_^2−^ → Cell-CaCO_3_ (7)

An *L. fusiformis* solution (LFS) for inoculation was prepared by streaking YA medium and incubating at 30 °C for 24 h to obtain single colonies, which were then inoculated into 10 mL of YB medium. The compositions of these media are presented in Table 2. For primary seeding, the inoculated single colonies were incubated at 30 °C and 180 rpm for 18 h. Next, a 2 L triflask was inoculated with 500 mL of YB medium and 10 mL of primary culture and then incubated at 30 °C and 180 rpm for 24 h. The cultured bacteria were transferred to sterilized centrifuge tubes and centrifuged at 7000 rpm for 1 min to remove the culture medium.

Cells were harvested from a total of 2.5 L of *L. fusiformis* culture. After removing the medium, the cells were washed once with saline solution and centrifuged again to remove any residual liquid. The final cell pellet was suspended in 1 L of tap water to prepare the LFS (1.87 × 10^8^ cells/mL).

### 2.2. Mixing Proportions and Sample Preparation

Table 3 shows the mixing proportions used in this study. The water–cement ratio was fixed at 50%, and 0%, 5%, 10%, 15%, and 20% of the water was replaced with the LFS. The range of mixing ratios that did not affect the final properties of the cement composite was explored in preliminary experiments to optimize the LFS mixing ratio, which led to a maximum mixing ratio of 20%. These mortars are hereafter referred to as the control, LFS05, LFS10, LFS15, and LFS20, respectively. To evaluate the compressive strength and compressive strength recovery rate of cement composites incorporating LFS, 50 × 50 × 50 mm cubes were fabricated. For the splitting tensile strength and carbonation depth tests, Ø50 × 100 mm cylindrical samples were fabricated, whereas Ø100 × 50 mm cylinders were fabricated to examine the crack healing performance.

The test samples were demolded 24 h after production and then cured in water at 20 °C until the required age.

The mortar flow and compressive strength were measured according to the KS L 5105 [41]. For the compressive strength recovery rate, the samples were subjected to a load of approximately 70% of the 28 d compressive strength, which was taken from the existing literature [42], followed by re-curing; then, the healing compressive strength was measured 7, 28, and 56 d after re-curing.

**Figure 1:**
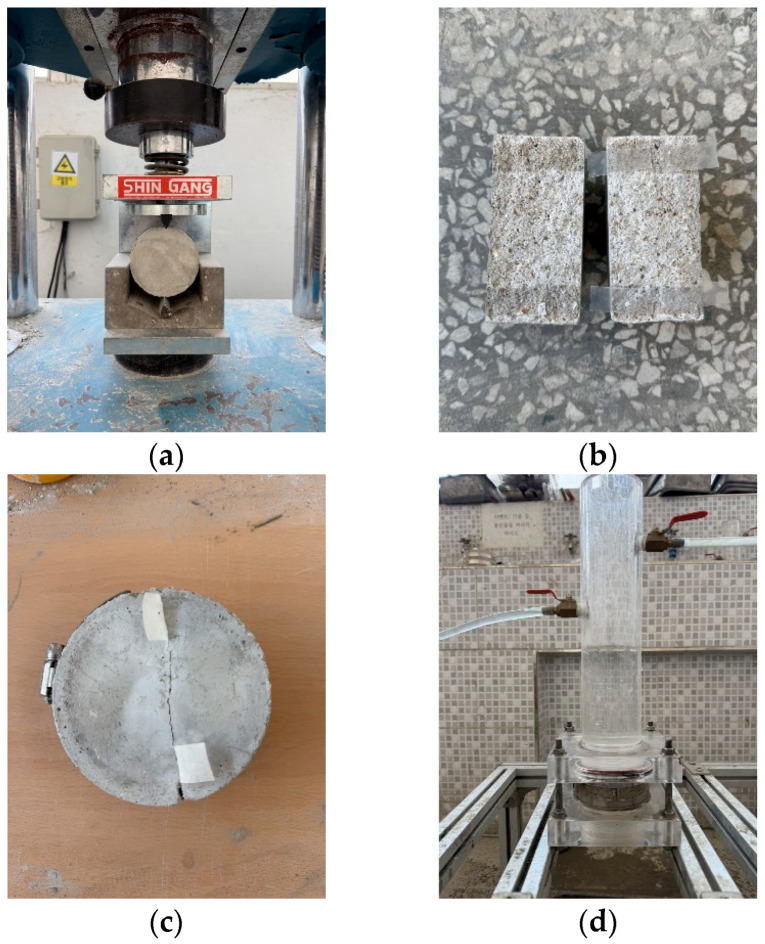
Water permeability testing: (**a**) splitting, (**b**) attaching a silicone sheet, (**c**) fastening the clamp, and (**d**) measuring the water passing through the crack.

The splitting tensile strength, carbonation depth, and healing ratio were measured in accordance with KS F 2423 [43] and KS F 2584 [44]. The healing ratio was measured according to KCI-CT 114 [45], and the laitance and foreign substances were removed from the cracked area after the sample was split at 28 d, as shown in Figure 1. To induce cracks, a silicone sheet was inserted into the side of the sample, and the sample was then fixed using a clamp. The crack width was fixed to approximately 0.2 mm, and measurements were taken at 7 d intervals until the healing age was 28 d.

The healing rate based on the measurement results was calculated as

(8) $$SHq=\left[1-\frac{q\left(t\right)}{q\left(0\right)}\right]\times 100(\%),$$

where *q*(*t*) represents the unit amount of water passing through the crack over time, and *q*(0) represents the unit amount of water passing through the crack on the first day of testing. In addition, *q*(*t*) was calculated by dividing the amount of water passed (mL) by the crack length (mm) and the measurement period (min), the unit being [mL/(min × mm)].

## 3. Results and Discussion

### 3.1. Characterizing the L. fusiformis Phenotype

**Figure 2:**
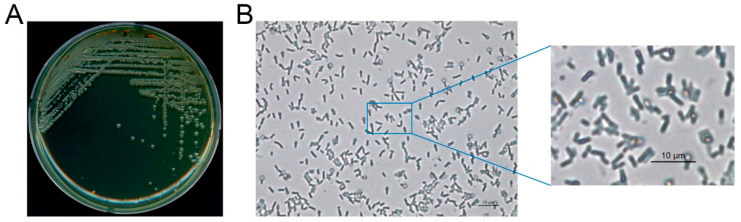
Phenotypic characteristics of the *L. fusiformis* used in this study. (**A**) Colonies of *L. fusiformis* grown on YA agar medium. (**B**) Cellular morphology of *L. fusiformis* observed by optical microscopy at 400× magnification, with a magnified view of the selected region.

**Figure 3:**
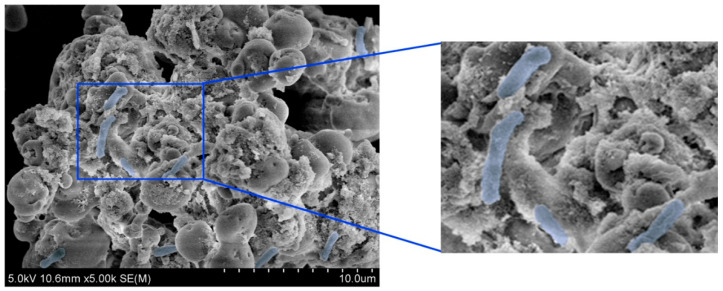
Field-emission scanning electron microscopy (FE-SEM) images of *L. fusiformis* at 5000× magnification. Bacterial cells (highlighted in blue) display a typical rod-shaped morphology.

The phenotypic and physiological characteristics of *L. fusiformis*, which was isolated from waste concrete, were examined prior to evaluating the self-healing performance of cement composites. As shown in Figure 2, optical microscopy revealed that this bacterial strain is composed of colonies and cells with typical morphologies. In addition, FE-SEM at 5000× magnification (Figure 3) revealed that the bacterial cells are rod-shaped. These preliminary results confirm that *L. fusiformis* is suitable for *use in* subsequent MICP experiments.

### 3.2. Mortar Flow

**Figure 4:**
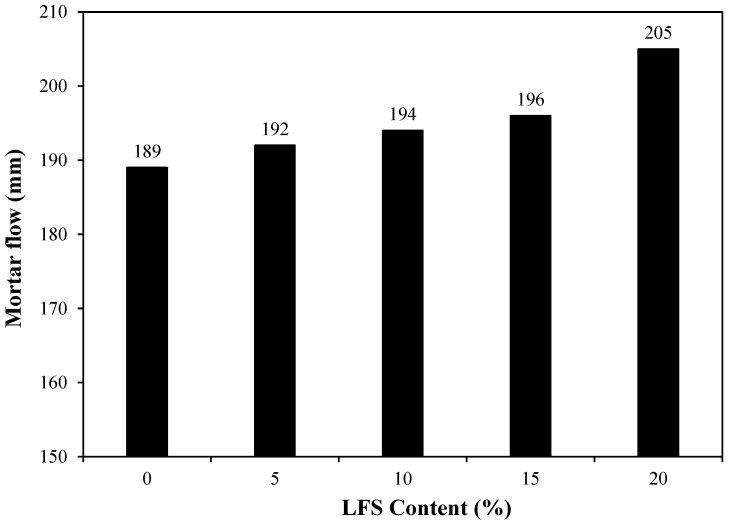
Mortar flow for various *L. fusiformis* solution (LFS) samples.

The effect of LFS on the mortar flow was examined, and the results are shown in Figure 4. Evidently, the mortar flow of the control sample was approximately 189 mm, which was the lowest among all samples. By comparison, the mortar flow of LFS05 increased by ~1.6% to ~192 mm. The mortar flow continued to increase with the increasing amount of LFS, and the LFS20 sample showed the highest flow of approximately 205 mm, approximately 8.5% higher than that of the control. The increase in the mortar flow with increasing LFS was attributed to the fact that the main component of the yeast extract used in the bacterial cultures was carbohydrates [46], which are sugar derivatives that act as surface active agents to increase mortar flow [47].

### 3.3. Compressive Strength

**Figure 5:**
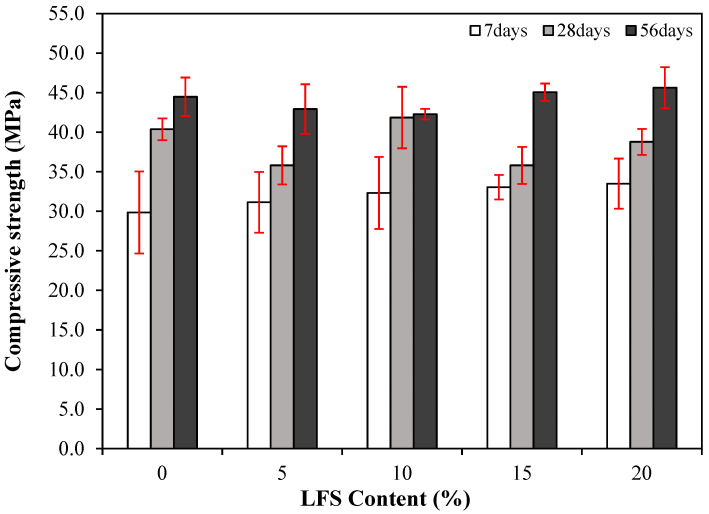
Changes in the compressive strength of the various LFS samples with age.

The effect of age on the compressive strength of mortar without and with various amounts of LFS is shown in Figure 5. The 7 d compressive strength of the control sample was the lowest at approximately 29.9 MPa, whereas that of LFS05 was relatively higher at ~31.1 MPa. In addition, the 7 d compressive strength generally increased as the amount of LFS increased, reaching approximately 33.5 MPa for LFS20, which was ~12% higher than that of the control sample. The relatively higher 7 d compressive strengths of the LFS-containing samples compared with that of the control samples are attributed to using the LFS as part of the mixing water. The LFS slightly increased the internal density owing to the bacteria it contained, as observed in previous studies [33].

The 28 d compressive strength of the control sample was approximately 40.4 MPa, which decreased slightly for LFS05 to approximately 35.8 MPa. Meanwhile, the 28 d compressive strength of LFS10 was ~41.9 MPa, which is approximately 3.7% higher than that of the control sample. Similar to LFS05, the 28 d compressive strength of LFS15 and LFS20 were also somewhat lower than that of the control sample. The generally similar or lower compressive strengths of the LFS-containing samples were attributed to the yeast extract in the medium, which contained a large amount of carbohydrates that retarded the silicate reaction [46].

Finally, the 56 d compressive strength of the control sample was approximately 44.5 MPa, whereas that of the LFS20 sample was the highest at approximately 45.6 MPa. The LFS15 sample also exhibited a relatively higher 56 d compressive strength (~45.1 MPa) compared with the control sample. Meanwhile, those of LFS05 and LFS10 were approximately 42.3 and 42.9 MPa, respectively, which were not significantly higher than those of the control sample, with differences of ~3.6% and ~4.9%.

**Figure 6:**
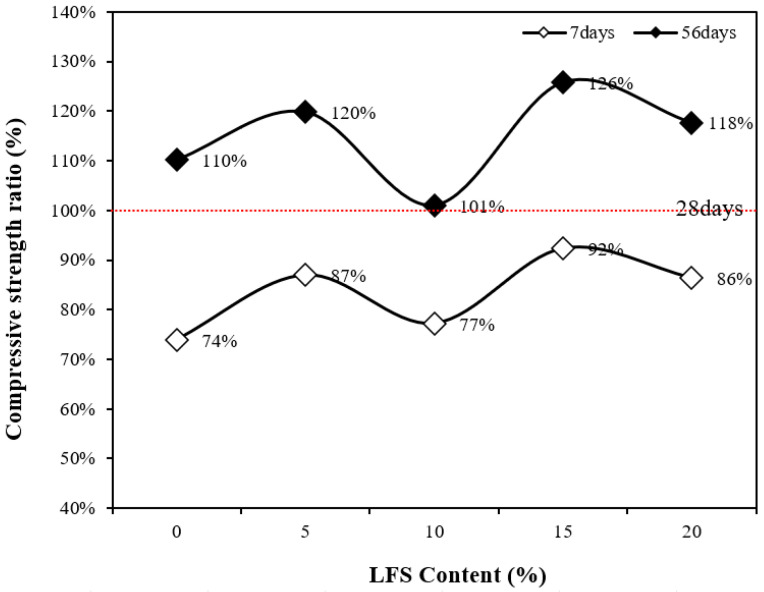
Compressive strength ratio as a function of the LFS content.

Figure 6 presents the compressive strength development rates at 7 and 56 d with respect to the 28 d compressive strength. Clearly, the compressive strength development rates at 56 d for the LFS-containing samples, except for LFS10, were relatively higher than those for the control sample. This difference is attributed to the silicate reaction, initially delayed by the presence of carbohydrates. However, this reaction then continued to progress over time, and as a result, the 56 d compressive strengths of the LFS15 and LSF20 samples were relatively higher than that of the control sample. In addition, although the development rate of the LFS10 sample was the lowest, its 28 d compressive strength was the highest at approximately 41.9 MPa. This higher reference point is hypothesized to be the reason for its lower development.

### 3.4. Splitting Tensile Strength

**Figure 7:**
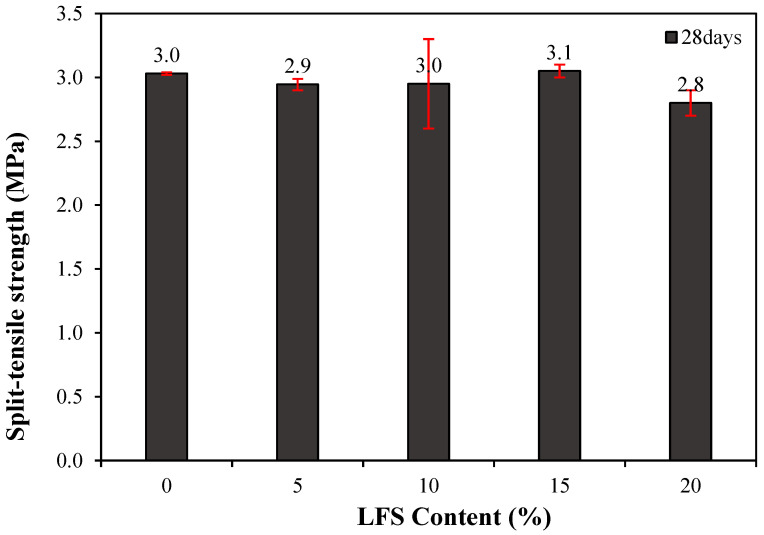
28 d splitting tensile strength of the mortar.

The 28 d splitting tensile strength of the mortar with and without LFS was also examined, as shown in Figure 7. The control sample exhibited a splitting tensile strength of approximately 3.0 MPa, whereas that of LFS05 was ~3.4% lower at ~2.9 MPa. However, as more LFS was incorporated, the splitting tensile strength tended to increase, reaching ~3.0 MPa for LFS10, which is similar to that of the control sample, and approximately 3.1 MPa for LFS15, the highest among the samples and ~3.3% higher than that of the control sample. By contrast, LFS20 demonstrated the lowest tensile strength of all samples at approximately 2.8 MPa, which is ~7.1% lower than the control sample.

### 3.5. Carbonation Resistance

**Figure 8:**
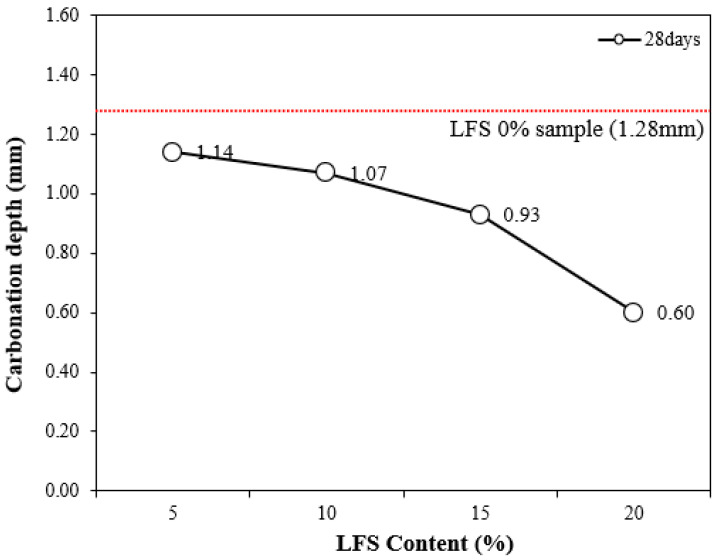
Carbonation depth at 28 d for various LFS contents.

Figure 8 shows the carbonation depth at 28 d of age as a function of the LFS content along with that of the control. The control sample had the highest carbonation depth of ~1.28 mm. By comparison, the carbonation depths of the LFS samples were lower, decreasing with the increasing incorporation of LFS. This is believed to be a result of the increased density because of the embedded bacteria [33].

In particular, the carbonation depth of LFS20 was approximately 0.60 mm, which was ~53% lower than that of the 0% LFS sample, implying the best carbonation resistance; this was also the sample with the highest 56 d compressive strength.

In general, bacteria-incorporated cement composites are known to inhibit an influx of CO_2_ because microcracks and voids are filled by the calcium carbonate formed by the MICP action of the bacteria [48]; similarly, this study has shown that the carbonation resistance increased with increasing LFS.

### 3.6. Compressive Strength Recovery Ratio

**Figure 9:**
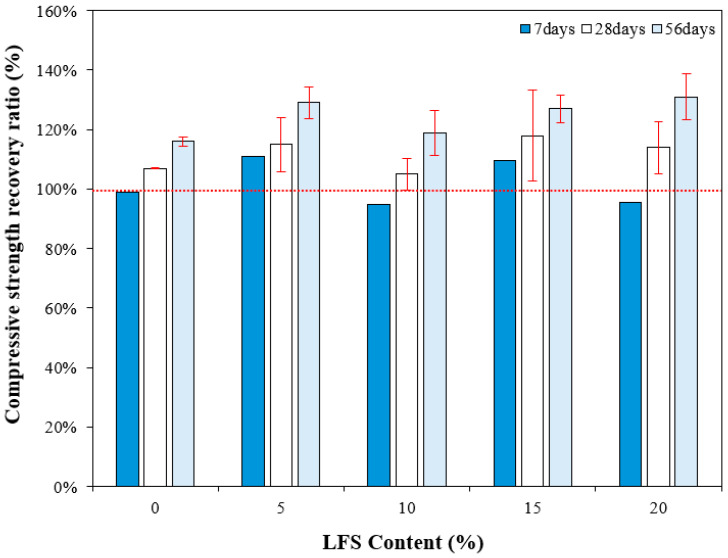
Compressive strength recovery ratio for various LFS contents after different periods of re-curing.

Figure 9 compares the 28 d compressive strength of mortar incorporating LFS and the compressive strength recovery rate according to the re-curing age. Evidently, the 7 d compressive strength recovery rate of the control sample after re-curing was approximately 99%. By contrast, those of the LFS05 and LFS15 were ~110–111%; thus, their compressive strengths after loading and then 7 d of re-curing exceeded the original 28 d compressive strength. However, the compressive strength recovery rates of the LFS10 and LFS20 samples were somewhat less than 100% of the 28 d compressive strength at this time, similar to the control sample.

After 28 d of re-curing, however, the compressive strength recovery rates of the control sample and LFS10 were similar at approximately 107% and 105%, respectively. The LFS05 and LFS15 samples, which showed excellent 7 d compressive strength recovery rates, continued to strengthen after 28 d of re-curing, with recovery rates of 115% and 118%, respectively. The compressive strength recovery rate was the highest in LFS15.

After 56 d of re-curing, the compressive strength recovery rate of the samples using LFS was approximately 119–131%, which is ~12.9% higher than that of the control sample (~116%), and the highest compressive strength recovery rate of approximately 131% was observed with LFS20. Thus, in the samples incorporating LFS, the healing performance improved with the increasing re-curing age, and the compressive strength recovery rate after 56 d of healing was better than that of the control sample. This result is attributed to the progressive bacterial metabolism, facilitated by the inflow of air and moisture through internal microcracks that formed when the load was applied [49].

### 3.7. Healing Ratio

**Figure 10:**
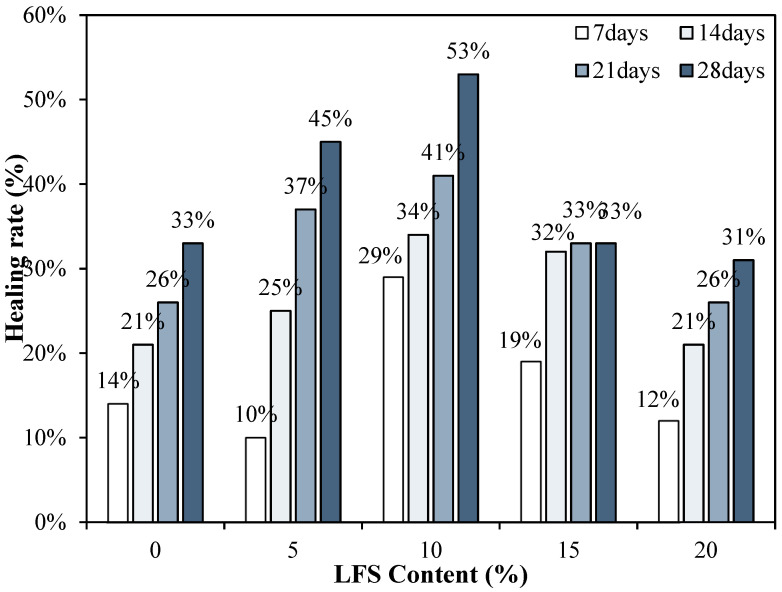
Healing rates for various LFS contents after different periods of re-curing.

Figure 10 shows the changes in the healing rate of the mortars using LFS. After re-curing for 7 d, the healing rate of the LFS10 sample was the highest at ~29%, and the healing rate of LFS05 was the lowest. Even after 14 d, the healing rate of LFS10 remained the highest at approximately 34%, whereas for LFS05, which had the lowest healing rate at 7 d, the healing rate was approximately 25%, which is ~4% higher than that of the control sample. In addition, the healing rate of LFS20 was approximately 21%, which is the lowest level among the LFS samples but similar to the control sample. After the final healing period of 28 d, LFS10 showed the best healing rate of approximately 53%.

**Figure 11:**
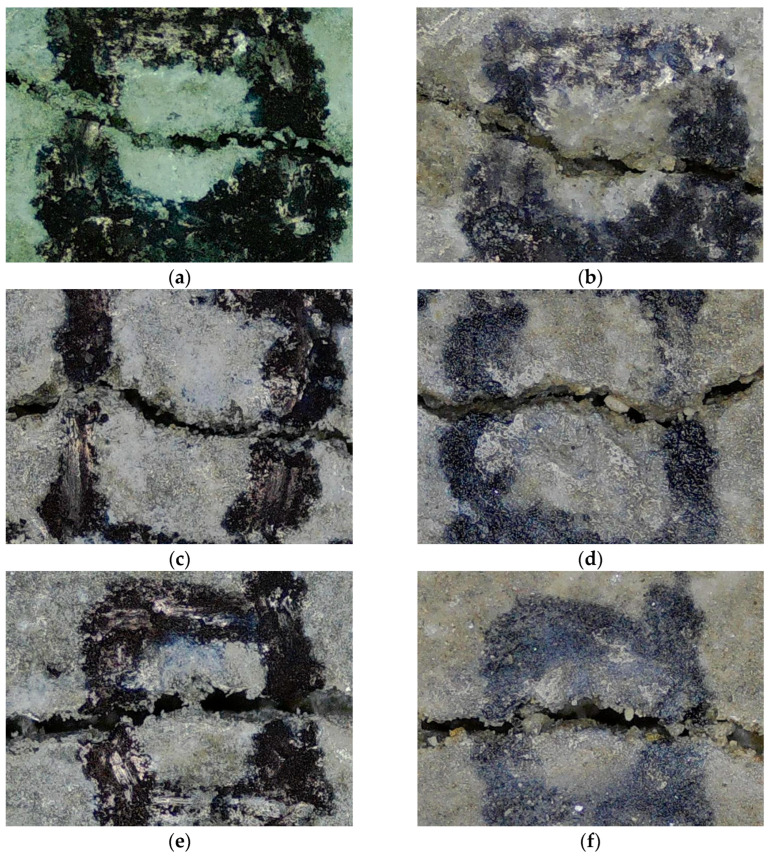
Crack width in mortars with 0% (control), 5%, 10%, 15%, and 20% LFS (labeled LFSx, where x is the LFS mixing ratio) at the time of cracking (0 d) and after 28 d of re-curing: (**a**) control 0 d, (**b**) control 28 d, (**c**) LFS10 0 d, (**d**) LFS10 28 d, (**e**) LFS20 0 d, and (**f**) LFS20 28 d.

Figure 11 displays crack-healing images for the control, LFS10, and LFS20 samples. The control sample exhibited no significant change in crack size even after 28 d of re-curing. By contrast, LFS10 exhibited a significantly more filled-in crack than the Control sample. Furthermore, in the LFS20 sample, which exhibited the poorest healing performance, the crack size decreased only marginally, consistent with the healing ratio results.

## 4. Conclusions

Based on the findings of this study, the following conclusions were obtained.

1. The mortar flow tended to increase with the increasing incorporation of LFS, which was likely because of the carbohydrate component in the bacterial medium acting as a surface active agent.
2. The compressive strength increased with the increasing LFS at the initial age of 7 d. The compressive strength development rate was evaluated on days 28 and 56, and the LFS samples showed relatively higher development rates than the control sample.
3. The splitting tensile strengths of the samples with and without LFS were ~2.8–3.1 MPa, showing no significant difference.
4. The carbonation depths showed that the carbonation resistance improved with the increasing incorporation of LFS, which was attributed to the incorporation of LFS, densifying the samples and metabolically producing hydrates that inhibited CO_2_ influx.
5. In the case of self-healing performance, the LFS20 showed the highest compressive strength recovery rate of approximately 131%, and in general, the compressive strength recovery rates of the samples incorporating LFS were superior to those of the control sample.

Considering the compressive strength development rates in this study, it is speculated that the compressive strength of the samples with LFS would likely continue to increase further with increasing age beyond 56 d compared with that of the control sample. Thus, additional research is required to evaluate the long-term compressive strength of the LFS-containing samples at ages exceeding 56 d.

## Figures and Tables

**Table 1:** Composition of the cement used in this study.

	SiO_2_ (wt%)	Al_2_O_3_ (wt%)	Fe_2_O_3_ (wt%)	CaO (wt%)	MgO (wt%)	K_2_O (wt%)	Blaine (cm^2^/g)	Density (g/cm^3^)
Ordinary Portland cement	17.43	6.50	3.57	64.4	2.55	1.17	3820	3.13

**Table 2:** Composition of the media used in this study.

Type	Reagent and Concentration (g/L)	
YA	Yeast extract	20
	Ammonium sulfate	10
	Ager	15
YB	Yeast extract	20
	Ammonium sulfate	10
PBS buffer (pH 7.4)	NaCl	8
	KCl	0.2
	Na_2_HPO_4_	1.44
	KH_2_PO_4_	0.24
BPU	Beef extract	3
	Peptone	5
	Urea	20
	Ager	15

**Table 3:** Mixing proportions of the mortars.

Type	LFS ^1^ (W × %)	LFS (g)	Water (g)	Cement (g)	Sand (g)
Cube (50 × 50 × 50 mm) Cylindrical (Ø50 × 100 mm) (Ø100 × 50 mm) Prismatic (40 × 40 × 160 mm)	0 5 10 15 20	0 52 103 155 206	1031 979 928 876 824	2062	4478

^1^ LFS: *L. fusiformis* solution.

## Data Availability

The original contributions presented in this study are included in the article. Further inquiries can be directed to the corresponding authors.

## Abbreviations

The following abbreviations are used in this manuscript:

MICP	microbially induced calcite precipitation
SEM	scanning electron microscopy
LFS	*L. fusiformis* solution
